# Supplementary material for: Multiplex precise base editing in cynomolgus monkeys
Source: Nat Commun. 2020 May 11;11:2325. doi: 10.1038/s41467-020-16173-0 (PMC7214463; doi:10.1038/s41467-020-16173-0)
Supplement: Supplementary file 8 — Supplementary Information [file 41467_2020_16173_MOESM8_ESM.pdf]

Multiplex precise base editing in cynomolgus monkeys

Zhang, Aida, del Rosario, Wilde *et al.*

Supplementary Information

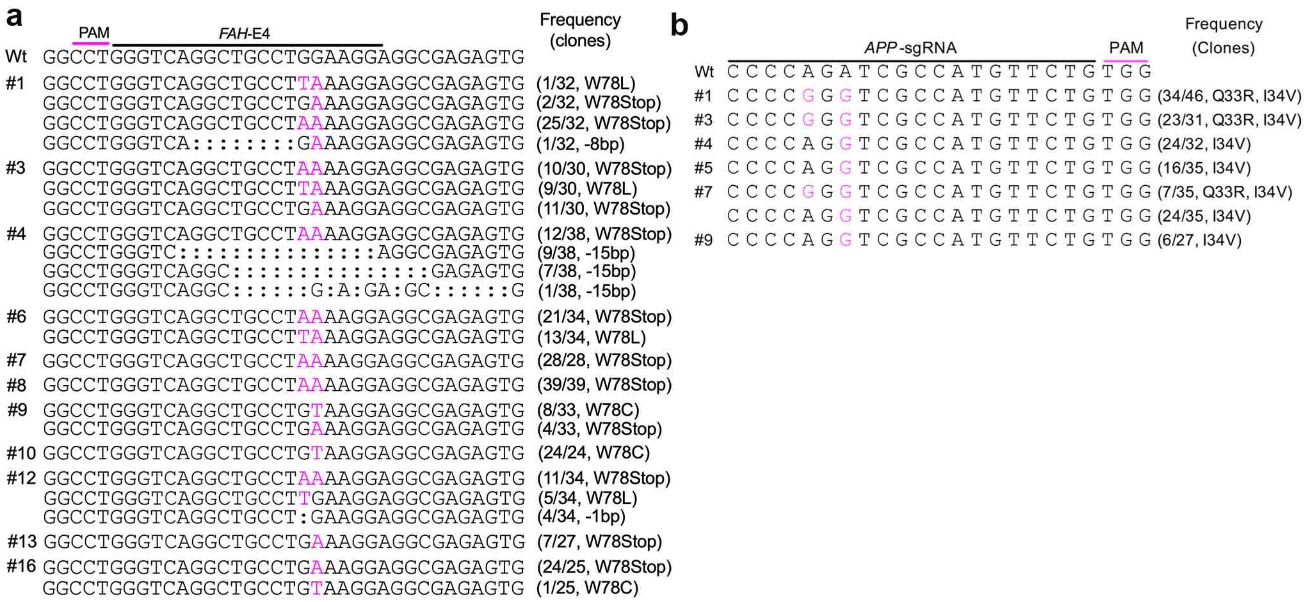

Supplementary Fig. 1 Single C-to-T or A-to-G base editing in monkey embryos. **a, b** Sanger sequence analysis of cloned (**a**) *FAH* exon 4 and (**b**) *APP* PCR amplicons. PAM and sgRNA target sequences are indicated by magenta and black lines, respectively. The converted nucleotides are shown in magenta letters. Wt: wild-type. Numbers in parentheses represent conversion-positive clones out of the total number of sequenced clones.

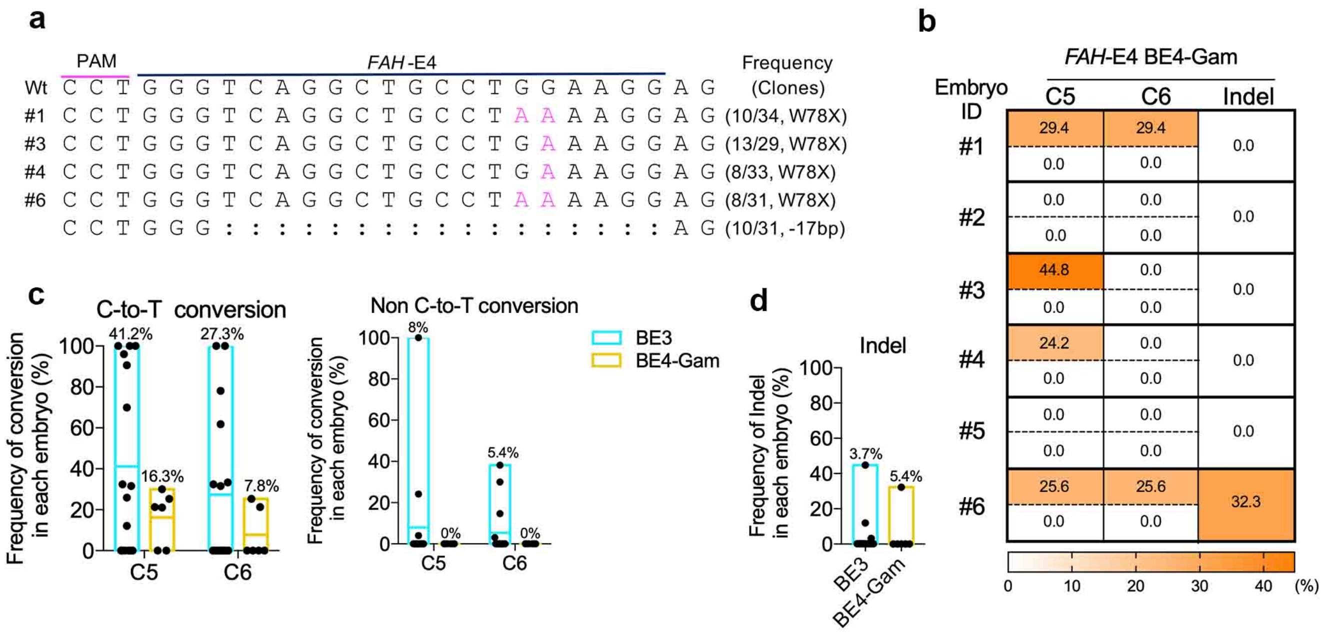

Embryo ID	C5	C6	Indel
#1	29.4	29.4	0.0
	0.0	0.0	
#2	0.0	0.0	0.0
	0.0	0.0	
#3	44.8	0.0	0.0
	0.0	0.0	
#4	24.2	0.0	0.0
	0.0	0.0	
#5	0.0	0.0	0.0
	0.0	0.0	
#6	25.6	25.6	32.3
	0.0	0.0	

Supplementary Fig. 2 BE4-Gam-mediated single C-to-T base editing in monkey embryos. **a** Sanger sequence analysis of cloned *FAH* exon 4 PCR amplicons by BE4-Gam. PAM and sgRNA target sequences are indicated as magenta and black lines, respectively. The converted nucleotides are shown in magenta letters. Wt: wild-type. Numbers in parentheses represent conversion-positive clones out of the total number of sequenced clones. **b** Base editing of *FAH* exon 4 by BE4-Gam. Numbers shown on the left of the table indicate embryo ID. In each embryo, top and bottom rows indicate intended C-to-T and unintended non-C-to-T conversions, respectively. **c** Efficiencies and accuracies of base editing by BE3 and BE4-Gam. Each dot represents one embryo. **d** Indel frequencies of base editing by BE3 or BE4-Gam. Each dot represents one embryo. In **c-d** data are represented as boxplots where the middle line is the mean (also shown as value on top of the bar), the top and bottom lines correspond to the maximum and minimum mutant allele frequencies. Each dot represents one embryo. BE3: Injections were performed thrice (total n = 16); BE4: Injections were performed twice (n = 6). Note that data of BE3 is same to Fig. 1.

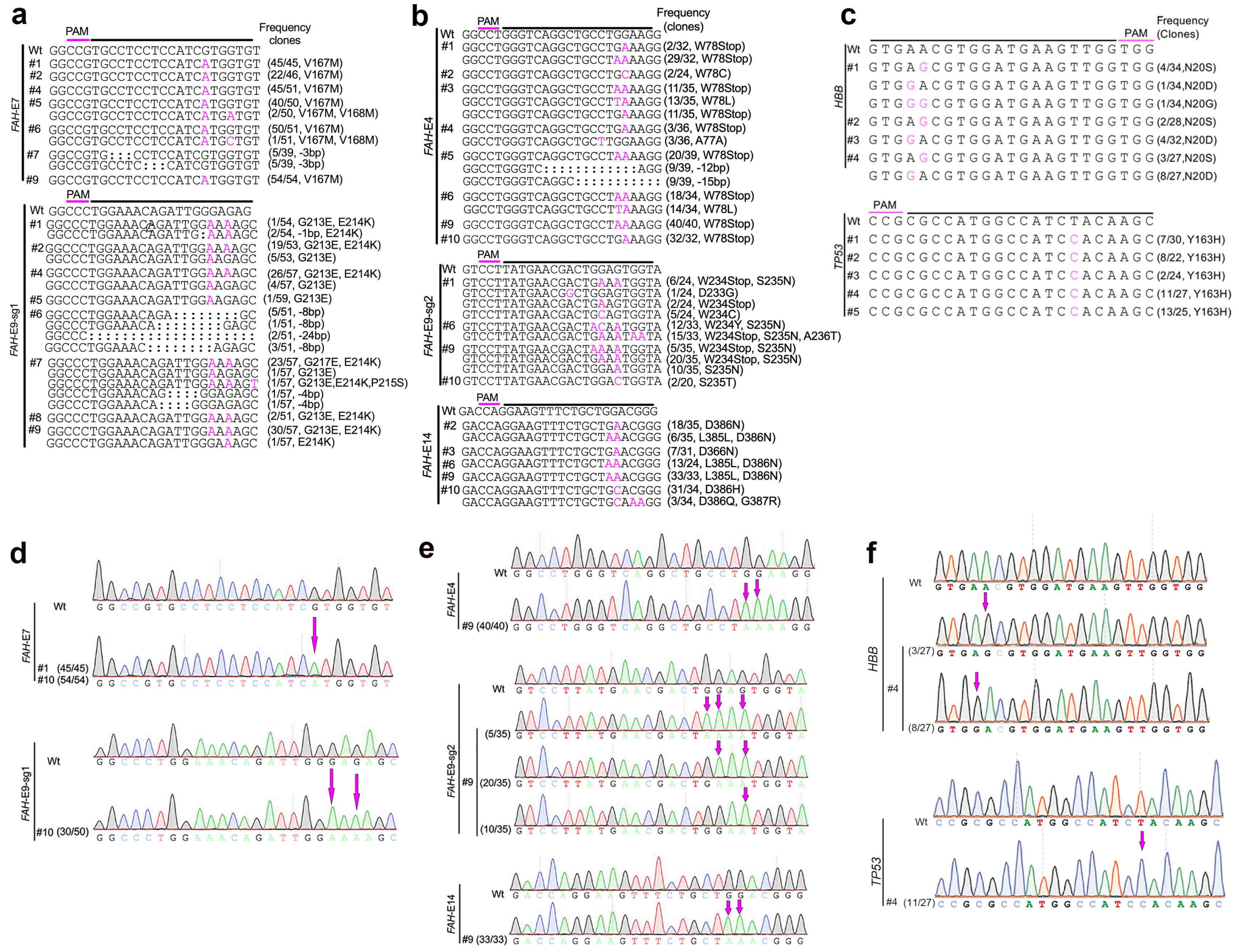

Supplementary Fig. 3 Double/triple multiplex C-to-T or A-to-G base editing in monkey embryos. **a-f** Sanger sequencing of cloned PCR products for (**a, d**) double or (**b, e**) triple multiplex C-to-T base editing by BE3 and (**c, f**) double multiplex and A-to-G base editing by ABE are shown. PAM and sgRNA target sequences are indicated by magenta and black lines, respectively. The converted nucleotides are shown as magenta letters. Wt: wild-type. Numbers in parentheses represent conversion-positive clones out of the total sequenced clones.

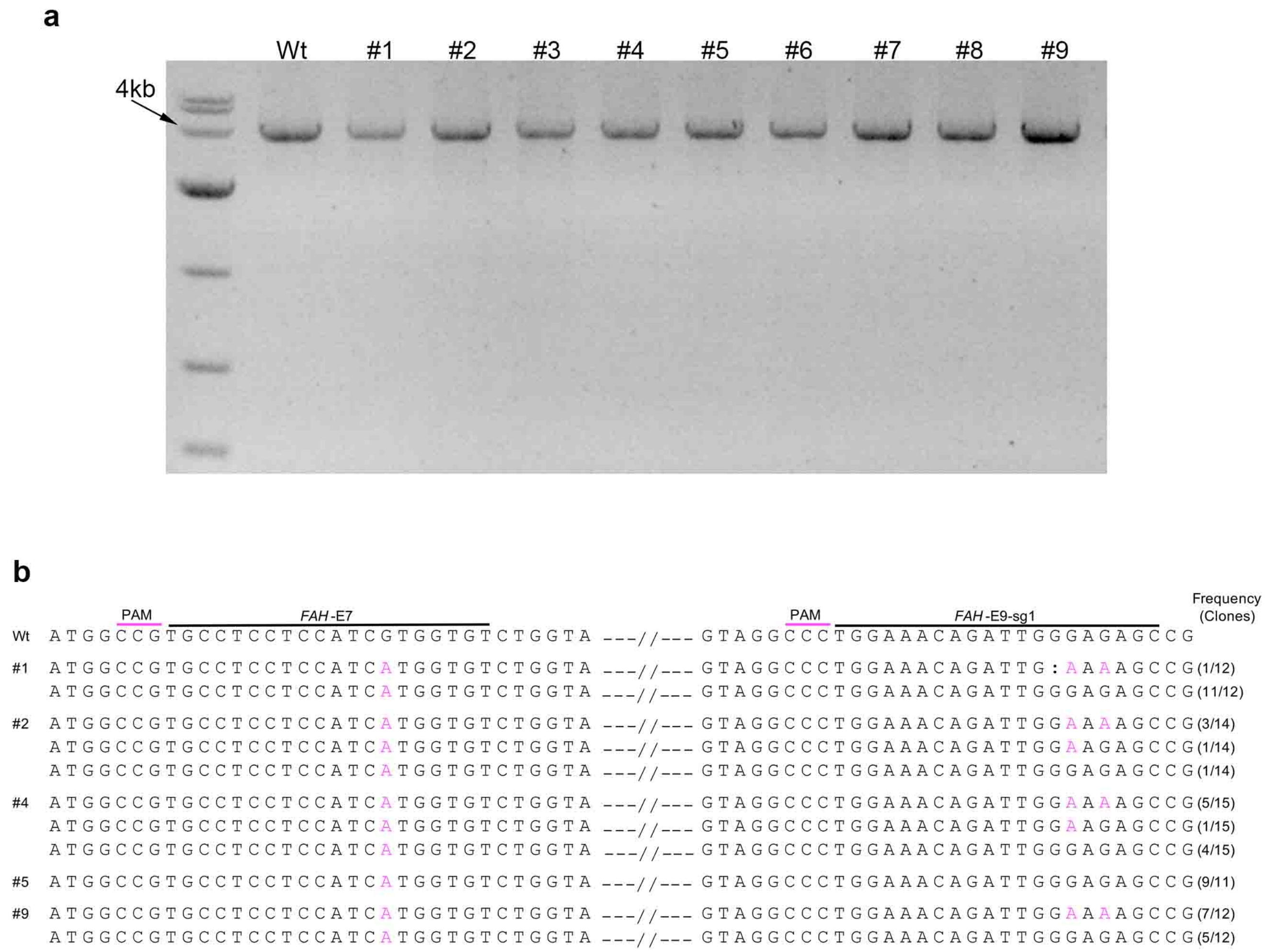

Supplementary Fig. 4 Double multiplex C-to-T base editing on the same *FAH* allele. **a** Long PCR across *FAH* exons 7 and 9 by PCR in each embryo subjected for double multiplex C-to-T base editing. **b** Sanger sequencing of cloned long PCR amplicons. PAM and sgRNA target sequences are indicated as magenta and black lines, respectively. The converted nucleotides are shown in magenta letters. Wt: wild-type. Numbers in parentheses represent conversion-positive clones out of the total number of sequenced clones.

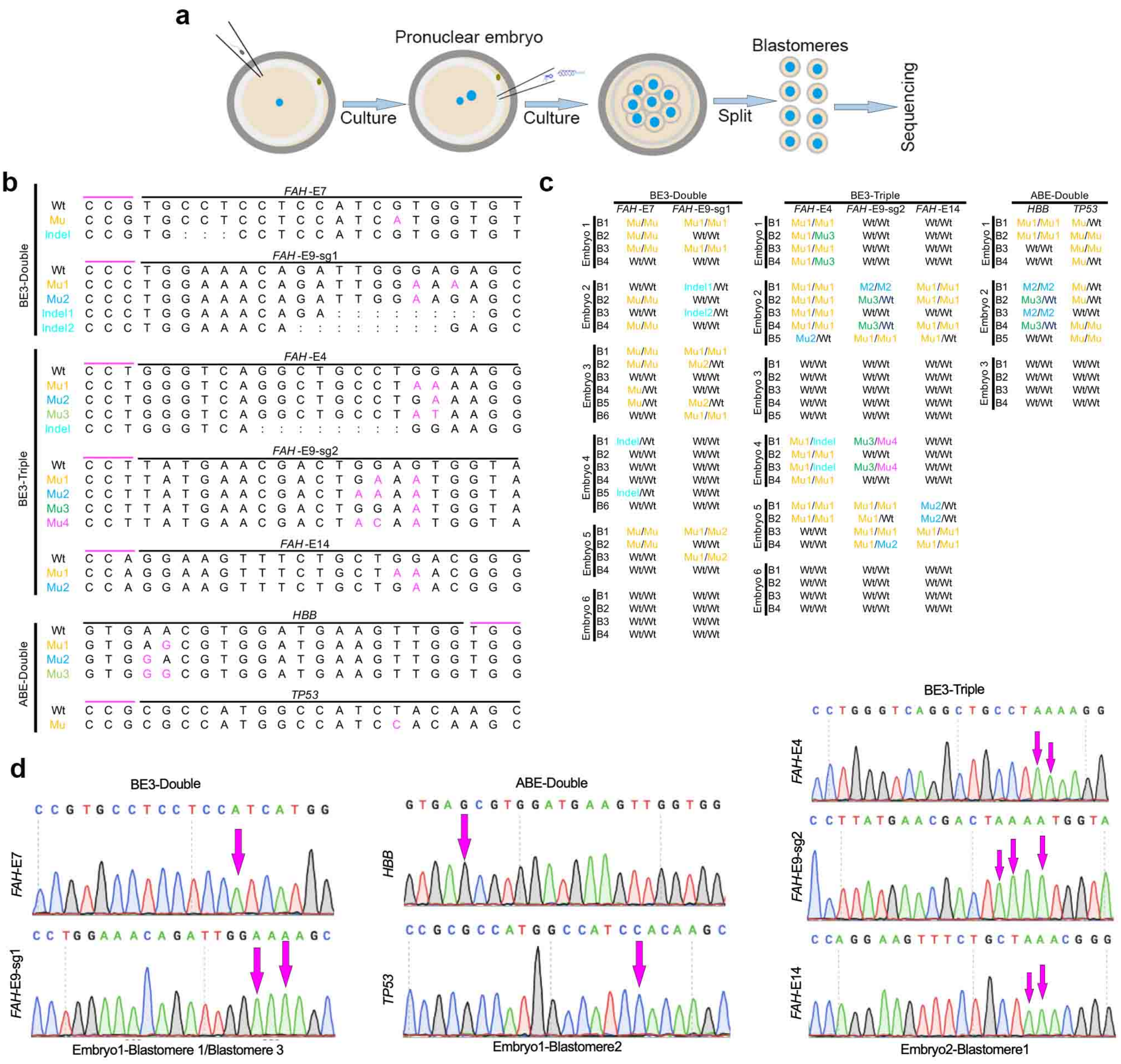

Supplementary Fig. 5 Single blastomere genotyping of double/triple multiplex C-to-T or A-to-G base editing in monkey embryos. **a** Schematic representation of single blastomere genotyping. **b** Modified alleles found in blastomeres. Converted nucleotides are shown in magenta. PAM and sgRNA target sequences are indicated by magenta and black lines, respectively. **c** Single blastomere genotyping. **d** Representative Sanger sequence chromatograms. Arrows indicate conversions.

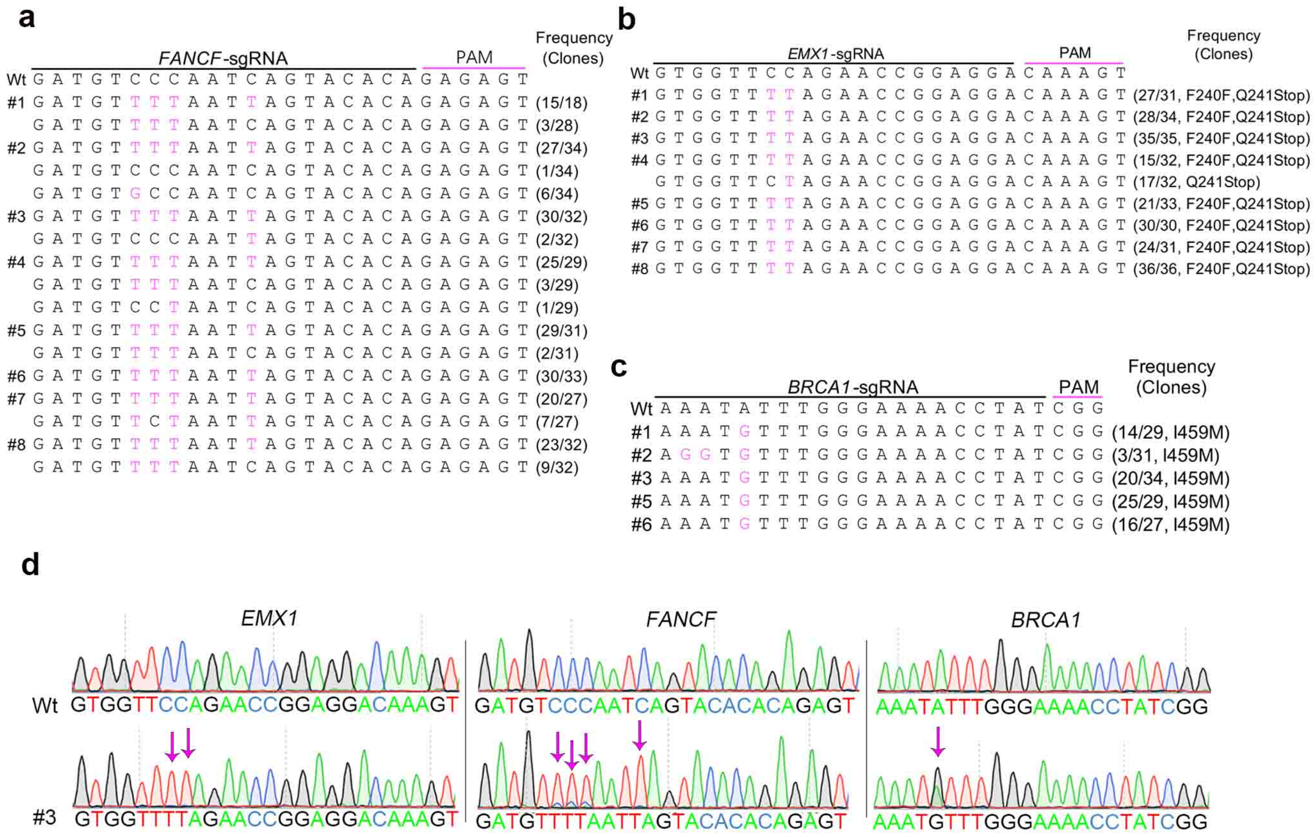

Supplementary Fig. 6 Triple multiplex C-to-T and A-to-G base editing in monkey embryos. **a-d** Sanger sequencing of cloned PCR amplicons for triple multiplex C-to-T and A-to-G base editing by (**a, b, d**) SaKKH-BE3 and (**c-d**) ABE are shown. PAM and sgRNA target sequences are indicated by magenta and black lines, respectively. The converted nucleotides are shown as magenta letters. Wt: wild-type. Numbers in parentheses represent conversion-positive clones out of the total number of sequenced clones.

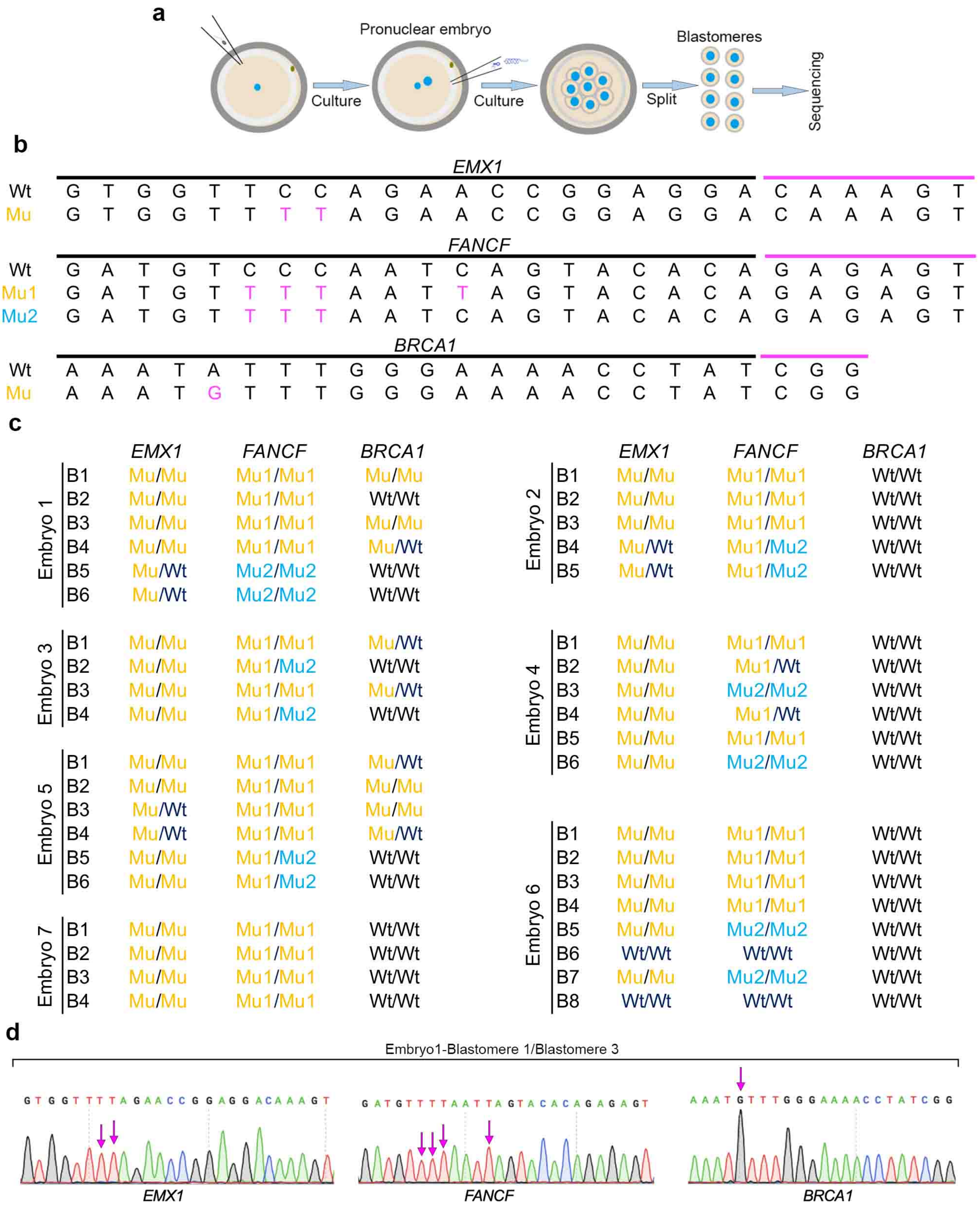

Supplementary Fig. 7 Single blastomere genotyping of triple multiplex C-to-T and A-to-G base editing in monkey embryos. **a** Schematic representation of single blastomere genotyping. **b** Modified alleles found in blastomeres. Converted nucleotides are shown in magenta. **c** Single blastomere genotyping. **d** Representative Sanger sequence chromatograms. Arrows indicate conversions.

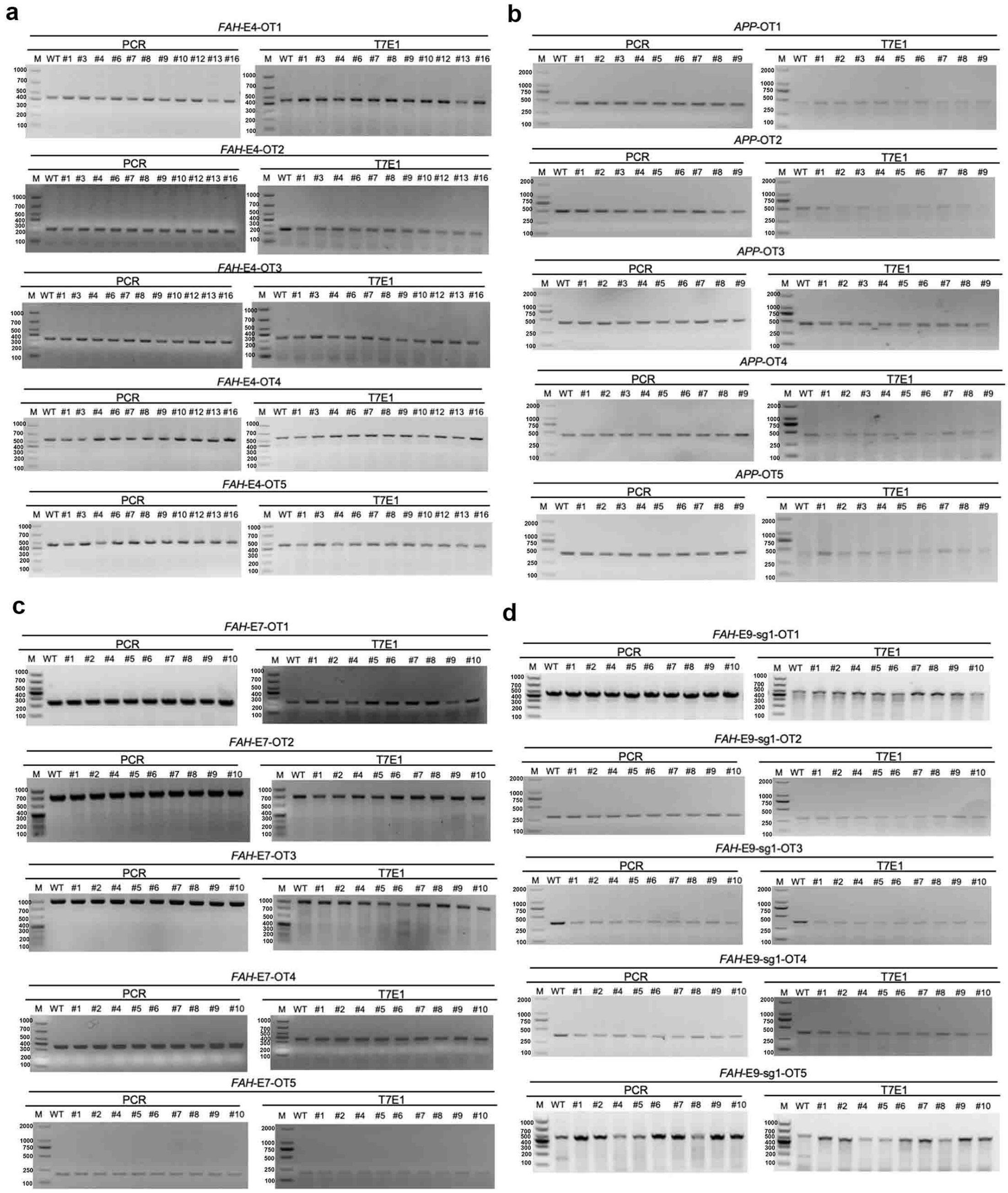
a
FAH-E4-OT1
PCR
T7E1
M WT #1 #3 #4 #6 #7 #8 #9 #10 #12 #13 #16
FAH-E4-OT2
FAH-E4-OT3
FAH-E4-OT4
FAH-E4-OT5
b
APP-OT1
M WT #1 #2 #3 #4 #5 #6 #7 #8 #9
APP-OT2
APP-OT3
APP-OT4
APP-OT5
c
FAH-E7-OT1
M WT #1 #2 #4 #5 #6 #7 #8 #9 #10
FAH-E7-OT2
FAH-E7-OT3
FAH-E7-OT4
FAH-E7-OT5
d
FAH-E9-sg1-OT1
FAH-E9-sg1-OT2
FAH-E9-sg1-OT3
FAH-E9-sg1-OT4
FAH-E9-sg1-OT5

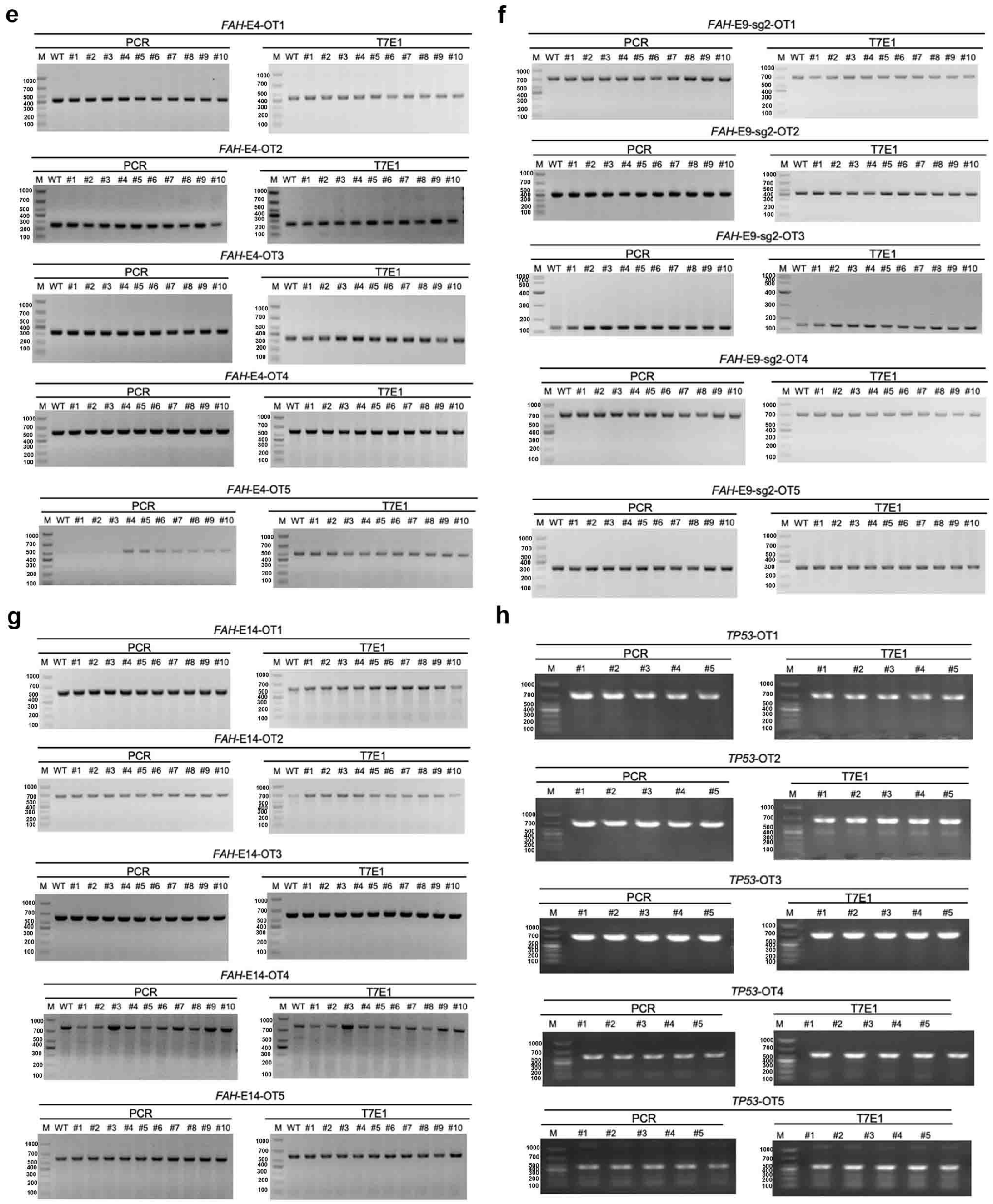

e
FAH-E4-OT1
PCR
T7E1
FAH-E4-OT2
PCR
T7E1
FAH-E4-OT3
PCR
T7E1
FAH-E4-OT4
PCR
T7E1
FAH-E4-OT5
PCR
T7E1
f
FAH-E9-sg2-OT1
PCR
T7E1
FAH-E9-sg2-OT2
PCR
T7E1
FAH-E9-sg2-OT3
PCR
T7E1
FAH-E9-sg2-OT4
PCR
T7E1
FAH-E9-sg2-OT5
PCR
T7E1
g
FAH-E14-OT1
PCR
T7E1
FAH-E14-OT2
PCR
T7E1
FAH-E14-OT3
PCR
T7E1
FAH-E14-OT4
PCR
T7E1
FAH-E14-OT5
PCR
T7E1
h
TP53-OT1
PCR
T7E1
TP53-OT2
PCR
T7E1
TP53-OT3
PCR
T7E1
TP53-OT4
PCR
T7E1
TP53-OT5
PCR
T7E1

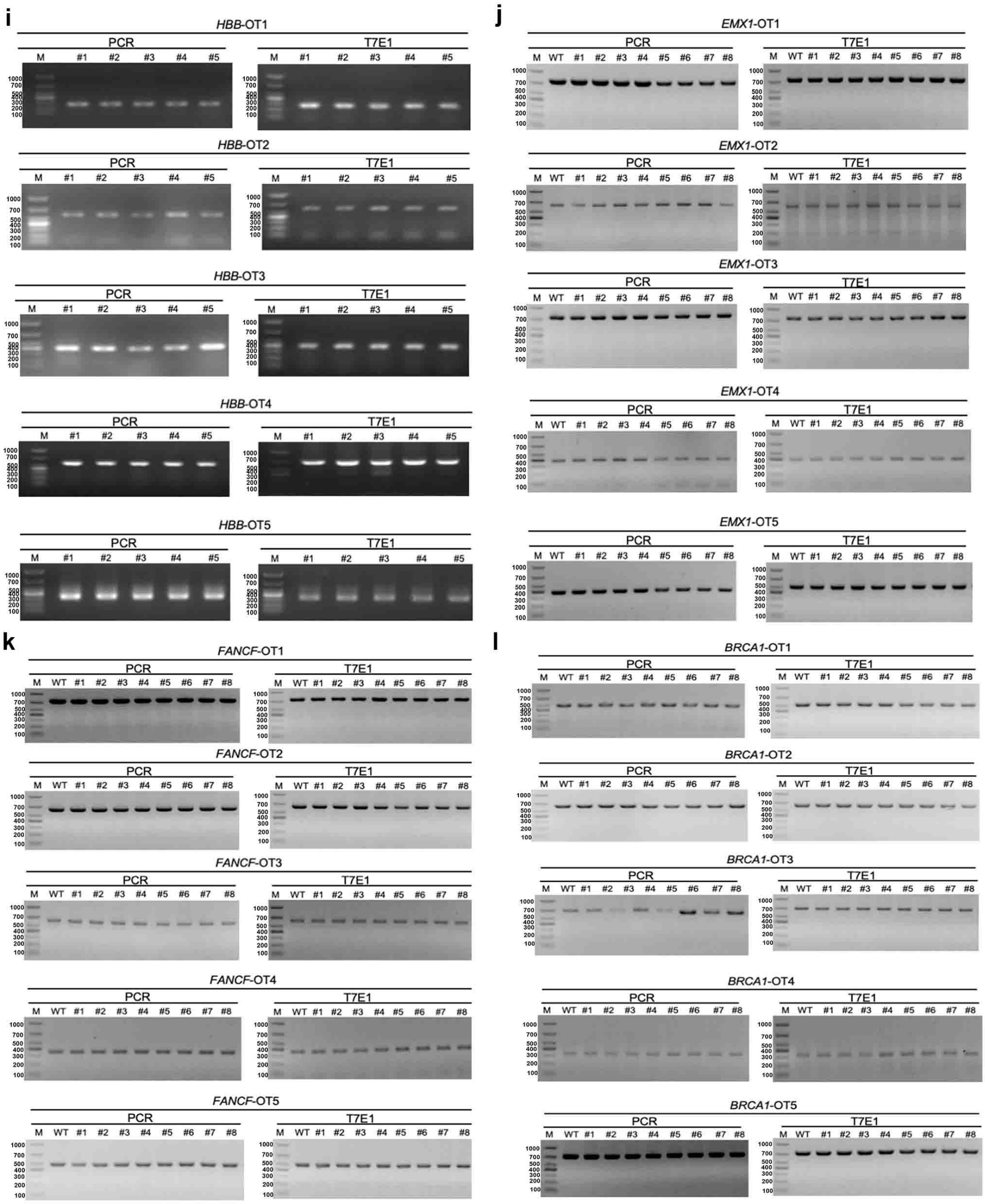

Supplementary Fig. 8 Targeted off-target analyses by T7E1. **a-i** T7E1 assays of potential off-target sites of (**a**) *FAH*-Exon4, (**b**) *APP*, (**c**)*FAH*-E7, (**d**) *FAH*-E9-sg1, (**e**) *FAH*-E4, (**f**) *FAH*-E9-sg2, (**g**) *FAH*-E14, (**h**) *TP53*, (**i**) *HBB*, (**j**) *FANCF*, (**k**) *EMX1* and (**l**) *BRCA1*. WT: wild-type, M: DNA size marker. Sizes are shown in left (bp). Source data of uncropped original gel images are provided as a Source Data file.

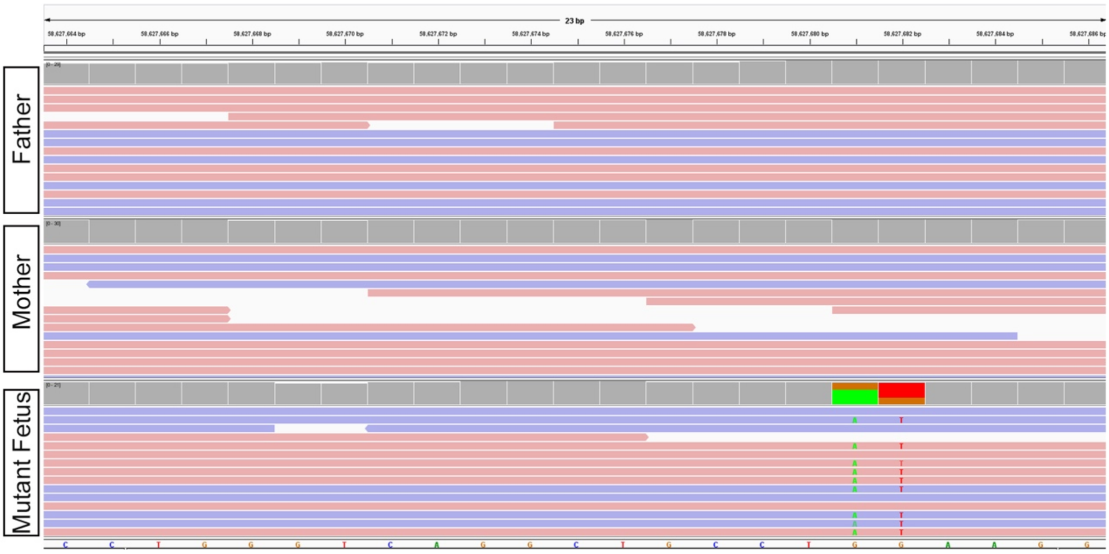

Supplementary Fig. 9 Trio-based whole genome sequencing of *FAH* mutant fetus. *FAH* on-target sites are shown. Pink and blue bars indicate forward and reverse sequencing reads, respectively. The nucleotides at conversion sites are shown as green for A and red for T for conversion, and brown for G for wild-type.

Supplementary Table 1 Genotyping control using wild-type embryos

Target site	Number of embryos analyzed	Number of clones sequenced	Number of clones with mutation
FAH-E4	6	58	0
FAH-E7		61	0
FAH-E9-sg1		56	0
FAH-E9-sg2		55	0
APP		62	0
HBB		61	0
TP53		56	0
EMX1		59	0
FANCF		58	0
BRCA1		57	0

Supplementary Table 2 BE4-Gam-mediated single C-to-T base editing in monkey embryos.

Editor	Target site	Analyzed	Edited	Indel	C>T	100% C>T
BE4-Gam	*FAH*	6	4 (66.7%)	1 (25%)	4 (100%)	0 (0%)

Injections were performed twice.

Supplementary Table 3 Targeted off-target analyses by T7E1 assay.

Base editor	Target sites	Number of off-target sites analyzed	Number of embryos analyzed	Number of embryos with off-target mutation
BE3-Single	*FAH*-E4	5	11	0
ABE-Single	*APP*	5	9	0
BE3-Double	*FAH* -E7	5	9	0
	FAH -E9-sg1	5		0
BE3-Triple	*FAH* -E4	5	10	0
	FAH -E9-sg2	5		0
	FAH -E14	5		0
ABE-Double	*HBB*	5	5	0
	TP53	5		0
SaKKHBE3/ ABE-Triple	*EMX1*	5	8	0
	FANCF	5		0
	TP53	5		0

Supplementary Table 4 Summary of embryo development.

Group	Injected	2-cell	8-cell	Morula	Blastocyst
Buffer	10	10 (100%)	8 (80%)	7 (70%)	5 (50%)
BE3-*FAH*	18	18 (100%)	16 (88.9%)	12 (66.7%)	7 (38.9%)

Injected: number of pronuclear stage embryos injected. Injections were performed thrice.

Supplementary Table 5 Summary of pregnancies.

Injected	Developed	Transferred	Surrogate mother	Pregnancy	Miscarriage	Modified
67	56	36	5	3	3	1

Injected: number of pronuclear stage embryos injected, Developed: number of embryos developed to 8-cell stage, Surrogate mother: number of surrogate mothers, Pregnancy: number of pregnant surrogate mothers, Miscarriage: number of miscarriages after amniotic fluid collection, Modified: number of miscarried fetus with *FAH* modification. Injections were performed thrice.

Supplementary Table 6 Estimation of genome-wide, guideRNA-independent off-target editing.

Substitution	dbSNP/EVA	Observed de novo	Expected de novo	Observed - Expected
A>C	72143	2	2.88	-0.88
A>G	241878	8	9.67	-1.67
A>T	55704	1	2.23	-1.23
C>A	69782	3	2.79	0.21
C>G	69523	6	2.78	3.22
C>T	317059	15	12.67	2.33
G>A	318389	14	12.72	1.28
G>C	69166	5	2.76	2.24
G>T	71360	3	2.85	0.15
T>A	55851	0	2.23	-2.23
T>C	239406	6	9.57	-3.57
T>G	71136	3	2.84	0.16

Supplementary Table 7 Primers for sgRNA synthesis

Name	Forward (5’ to 3’)	Reverse (5’ to 3’)
FAH-E4-sgRNA	TAATACGACTCACTATAGCCTTCCAGGCAGCCTGACCCGTTTTAGAGCTAGAAATAGC	AGCACCGACTCGGTGCCACTT
FAH-E7-sgRNA	TAATACGACTCACTATAGACACCACGATGGAGGAGGCAGTTTTAGAGCTAGAAATAGC	AGCACCGACTCGGTGCCACTT
FAH-E9-sgRNA1	TAATACGACTCACTATAGTACCACTCCAGTCGTTCATAGTTTTAGAGCTAGAAATAGC	AGCACCGACTCGGTGCCACTT
FAH-E9-sgRNA2	TAATACGACTCACTATAGGCTCTCCCAATCTGTTTCCAGTTTTAGAGCTAGAAATAGC	AGCACCGACTCGGTGCCACTT
FAH-E14-sgRNA	TAATACGACTCACTATAGCCCGTCCAGCAGAAACTTCCGTTTTAGAGCTAGAAATAGC	AGCACCGACTCGGTGCCACTT
APP-SgRNA	TAATACGACTCACTATAGCCCCAGATCGCCATGTTCTGGTTTTAGAGCTAGAAATAGC	AGCACCGACTCGGTGCCACTT
HBB-SgRNA	TAATACGACTCACTATAGGTGAACGTGGATGAAGTTGGGTTTTAGAGCTAGAAATAGC	AGCACCGACTCGGTGCCACTT
TP53-SgRNA	TAATACGACTCACTATAGGCTTGTAGATGGCCATGGCGGTTTTAGAGCTAGAAATAGC	AGCACCGACTCGGTGCCACTT
BRCA1-SgRNA	TAATACGACTCACTATAGAAATATTTGGGAAAACCTATGTTTTAGAGCTAGAAATAGC	AGCACCGACTCGGTGCCACTT
EMX1-SgRNA	TAATACGACTCACTATAGGTGGTTCCAGAACCGGAGGAGTTTTAGTACTCTGTAATGA	AAAAAAATCTCGCCAACAAGTTGACGA
FANCF-SgRNA	TAATACGACTCACTATAGGATGTCCCAATCAGTACACAGTTTTAGTACTCTGTAATGA	AAAAAAATCTCGCCAACAAGTTGACGA

Supplementary Table 8 Primer for on-target amplification

Name	Forward (5' to 3')	Reverse (5' to 3')
FAH-E4	GCCAAGTTTAGCACAGTCGC	GGGAGCAGGTCTCACTTGAC
FAH-E7	CCACTGCACCTAGTCCAGAA	TCCAGTTCCATGTCCAAGAGC
FAH-E9	CAGTCCTGATCCATGGCTGG	TTATGGTGTCAGCCGTGTCC
FAH-E14	GGAAGAAGGGGCAGAGTTCC	ACTCACGCTTGAAGTCCTGG
APP	ATAGAGCTCTTTTTGATCCCTGTT	CAGGTTTTGGTCCCTGATGGAT
HBB	TGGCTGTCATCACTTAGACC	CATCACTAAAGGCACCAAGCA
TP53	CTAGCTGCCTAGTGGGTTGC	CGGATAAGGTGCTGAGGAGG
EMX1	GGGGGCCCTAACCCTATGTA	AGGGAGATTGGAGACACGGA
FANCF	ACATCTGCTCTCCCTCCACT	GGACATCACCATGACCGAGG
BRCA1	GGGTCTGAATCAAATGCCAAAGT	TTATTTGTGAGGGGACGCTCT
FAH-E4-deep	GAAGTGATTACGGTGTGCTCCCATTGGCAGGAGGGATA	GAGTTGGATGCTGGATGGTCAGAATGGCACTGACCACC
