# Supplementary material for: Multiplex precise base editing in cynomolgus monkeys
Source: Nat Commun. 2020 May 11;11:2325. doi: 10.1038/s41467-020-16173-0 (PMC7214463; doi:10.1038/s41467-020-16173-0)
Supplement: Supplementary file 9 — Reporting Summary [file 41467_2020_16173_MOESM9_ESM.pdf]

nature research

Corresponding author(s): DBPR and NCOMMS-18-34407B

Last updated by author(s): Apr 4, 2020

Reporting Summary

Nature Research wishes to improve the reproducibility of the work that we publish. This form provides structure for consistency and transparency in reporting. For further information on Nature Research policies, see Authors & Referees and the Editorial Policy Checklist .

Statistics

For all statistical analyses, confirm that the following items are present in the figure legend, table legend, main text, or Methods section.

n/a	Confirmed	
☐	☒	The exact sample size (*n*) for each experimental group/condition, given as a discrete number and unit of measurement
☐	☒	A statement on whether measurements were taken from distinct samples or whether the same sample was measured repeatedly
☐	☒	The statistical test(s) used AND whether they are one- or two-sided<br>*Only common tests should be described solely by name; describe more complex techniques in the Methods section.*
☒	☐	A description of all covariates tested
☒	☐	A description of any assumptions or corrections, such as tests of normality and adjustment for multiple comparisons
☐	☒	A full description of the statistical parameters including central tendency (e.g. means) or other basic estimates (e.g. regression coefficient) AND variation (e.g. standard deviation) or associated estimates of uncertainty (e.g. confidence intervals)
☒	☐	For null hypothesis testing, the test statistic (e.g. *F*, *t*, *r*) with confidence intervals, effect sizes, degrees of freedom and *P* value noted<br>*Give P values as exact values whenever suitable.*
☒	☐	For Bayesian analysis, information on the choice of priors and Markov chain Monte Carlo settings
☒	☐	For hierarchical and complex designs, identification of the appropriate level for tests and full reporting of outcomes
☒	☐	Estimates of effect sizes (e.g. Cohen's *d*, Pearson's *r*), indicating how they were calculated

Our web collection on statistics for biologists contains articles on many of the points above.

Software and code

Policy information about availability of computer code

Data collection	SnapGene (version 4.1.8) was used for collecting sequencing data.
Data analysis	bwa-mem (version 0.7.15-r1140), Macaca fascicularis genome assembly GCF_000364345.1), PicardTools (version 1.111(1909), GATK (version gatk-4.1.3.0) and IGV (version 2.6.1) were used for analysis of whole genome sequencing. Graphpad prism 7 was used for statistical analysis and graph production.

For manuscripts utilizing custom algorithms or software that are central to the research but not yet described in published literature, software must be made available to editors/reviewers. We strongly encourage code deposition in a community repository (e.g. GitHub). See the Nature Research guidelines for submitting code & software for further information.

Data

Policy information about availability of data

All manuscripts must include a data availability statement. This statement should provide the following information, where applicable:
- Accession codes, unique identifiers, or web links for publicly available datasets
- A list of figures that have associated raw data
- A description of any restrictions on data availability

All other data supporting the findings of this study are available within the paper and the associated supplementary files. The raw data of whole genome sequencing and deep sequencing will be deposited to NCBI Sequence Read Archive database (SRA) under accession code PRJNA505503 and PRJNA561611.

Field-specific reporting

Please select the one below that is the best fit for your research. If you are not sure, read the appropriate sections before making your selection.

Life sciences study design

All studies must disclose on these points even when the disclosure is negative.

Sample size	No methods were used to predetermine sample size. Sample sizes were chosen due to being able to show reproducibility, similar to those reported in previous publications of genome editing in monkey embryos (Niu et al., Cell 2014; Liu et al., Cell Stem Cell 2014), based on our editing efficiencies.
Data exclusions	No data were excluded from the analyses.
Replication	For each target site, 5-16 embryos from 2-3 independent injections were tested. All injections gave similar results.
Randomization	The fertilized embryos were randomly assign to each injection.
Blinding	Injection and analysis were performed by different experimenters. Injection mixture were blinded.

Reporting for specific materials, systems and methods

We require information from authors about some types of materials, experimental systems and methods used in many studies. Here, indicate whether each material, system or method listed is relevant to your study. If you are not sure if a list item applies to your research, read the appropriate section before selecting a response.

Materials & experimental systems

n/a	Involved in the study	
☒	☐	Antibodies
☒	☐	Eukaryotic cell lines
☒	☐	Palaeontology
☐	☒	Animals and other organisms
☒	☐	Human research participants
☒	☐	Clinical data

Methods

n/a	Involved in the study	
☒	☐	ChIP-seq
☒	☐	Flow cytometry
☒	☐	MRI-based neuroimaging

Animals and other organisms

Policy information about studies involving animals; ARRIVE guidelines recommended for reporting animal research

Laboratory animals	Cynomolgus monkeys are raised at Guangdong Landao Biotechnology Co. Ltd (Landao), which has the Association for Assessment and Accreditation of Laboratory Animal Care accredited facility. Eleven years old male monkey and 5-9 years old female monkeys were used.
Wild animals	No wild animals were used in this study.
Field-collected samples	No field-collected samples were used in this study.
Ethics oversight	All animal protocols were approved in advance by the Institutional Animal Care and Use Committee of Guangdong Landao Biotechnology Co. Ltd and South China Agricultural University.

Note that full information on the approval of the study protocol must also be provided in the manuscript.
