# Supplementary material for: Multiplex precise base editing in cynomolgus monkeys
Source: Nat Commun. 2020 May 11;11:2325. doi: 10.1038/s41467-020-16173-0 (PMC7214463; doi:10.1038/s41467-020-16173-0)
Supplement: Supplementary file 10 — Description of Additional Supplementary Files [file 41467_2020_16173_MOESM10_ESM.pdf]

Title: Supplementary Data 1:

Description: Off-target sites

Title: Supplementary Data 2:

Description: Primers for off-target site amplification

Title: Supplementary Data 3:

Description: *de novo* SNVs and indels by WGS

Title: Supplementary Data 4:

Description: Off-target sites predicted by Benchling

Title: Supplementary Data 5:

Description: Off-target sites predicted by CRISPOR

Title: Supplementary Data 6:

Description: Off-target sites predicted by Cas-OFFinder
